# Hypergraph Semi-Supervised Contrastive Learning for Hyperedge Prediction Based on Enhanced Attention Aggregator

**DOI:** 10.3390/e27101046

**Published:** 2025-10-08

**Authors:** Hanyu Xie, Changjian Song, Hao Shao, Lunwen Wang

**Affiliations:** College of Electronic Engineering, National University of Defense Technology, Hefei 230031, China; xiehanyu19@nudt.edu.cn (H.X.); songchangjian@nudt.edu.cn (C.S.); shaohao@nudt.edu.cn (H.S.)

**Keywords:** hyperedge prediction, hypergraph attention networks, order propagation, semi-supervised learning, key node augmentation

## Abstract

Hyperedge prediction is crucial for uncovering higher-order relationships in complex systems but faces core challenges, including unmodeled node influence heterogeneity, overlooked hyperedge order effects, and data sparsity. This paper proposes Order propagation Fusion Self-supervised learning for Hyperedge prediction (OFSH) to address these issues. OFSH introduces a hyperedge order propagation mechanism that dynamically learns node importance weights and groups neighbor hyperedges by order, applying max–min pooling to amplify feature distinctions. To mitigate data sparsity, OFSH incorporates a key node-guided augmentation strategy with adaptive masking, preserving core high-order semantics. It identifies topological hub nodes based on their comprehensive influence and employs adaptive masking probabilities to generate augmented views preserving core high-order semantics. Finally, a triadic contrastive loss is employed to maximize cross-view consistency and capture invariant semantic information under perturbations. Extensive experiments on five public real-world hypergraph datasets demonstrate significant improvements over state-of-the-art methods in AUROC and AP.

## 1. Introduction

**Figure 1:**
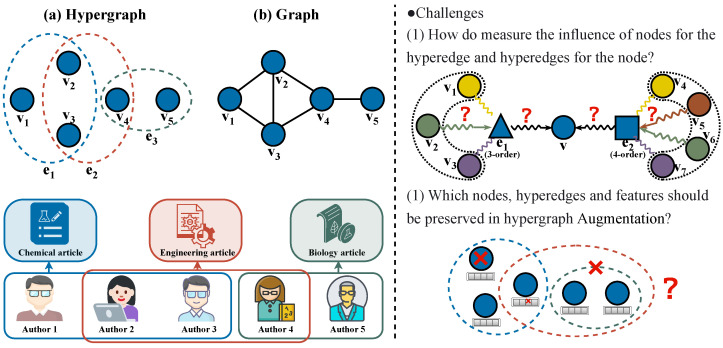
The collaborative relationships in academic networks are modeled as (**a**) a hypergraph and (**b**) a conventional graph. The right half of the figure illustrates the key challenges in hyperedge prediction.

Graphs have been extensively employed for modeling real-world networks [1], finding widespread applications in diverse domains including social network analysis [2], bioinformatics [3], and knowledge bases [4]. Nevertheless, the complexity of real-world relational systems often exceeds the expressive power of traditional graph models, which are confined to capturing only pairwise relationships between nodes, whereas higher-order group-wise interactions are prevalent. Representative examples include the following: (1) research collaboration groups co-authoring papers in academic networks, (2) user clusters engaging in email conversation threads within social networks, and (3) chemical reactions collectively catalyzed by groups of compounds in protein interaction networks. Modeling such group relationships using conventional graphs may result in significant semantic information loss. As demonstrated in Figure 1a, in the collaborative network scenario, authors $v_{1}$, $v_{2}$, and $v_{3}$ participate in a group-wise interaction by jointly completing a paper. However, the traditional graph representation shown in Figure 1b can merely depict potential pairwise collaborations among them, thereby failing to explicitly capture the specific higher-order relationship of their triadic co-authorship on a particular paper.

Distinct from ordinary graphs, hypergraphs serve as a more generalized graph structure that can naturally represent group-wise (higher-order) relationships involving arbitrary numbers of nodes through hyperedges, thereby effectively avoiding information loss and demonstrating advantages in modeling complex real-world systems. Consequently, network representation learning methods on hypergraphs [5,6,7,8,9,10] have been extensively studied, supporting various downstream tasks, including node classification [11], node ranking [12], link prediction [13], and—the key focus of this work—hyperedge prediction [14].

The hyperedge prediction task involves inferring the existence of unobserved or future hyperedges from the observed hypergraph structure. Common application scenarios include predicting groups of users who may co-purchase specific products [15] or identifying sets of proteins that might jointly catalyze chemical reactions [16]. This prediction process typically consists of two key steps: (1) generating node embeddings and (2) scoring candidate hyperedges. Traditional approaches mainly employ hypergraph neural networks (HGNNs) [17,18,19,20,21,22,23,24] to train supervised learning models. These models generally follow a binary classification framework that classifies potential hyperedges as either positive (existing) or negative (non-existing) samples before making predictions based on the learned hyperedge embeddings. However, since the number of potential hyperedges grows exponentially ($O(2^{n})$), observed real hyperedges become extremely sparse in the search space, creating significant challenges for generating effective negative samples during supervised learning. To overcome this challenge, state-of-the-art models often incorporate contrastive learning (CL) mechanisms [25,26,27], while sophisticated negative sampling strategies [8,28] have also demonstrated their importance in learning effective hypergraph structural representations.

Despite numerous methodological advancements, the fundamental challenges in hyperedge prediction remain underexplored.

***Challenge I: Node Aggregation for Hyperedge Prediction.** “How can we effectively capture the complex interactions among nodes as well as between nodes and hyperedges?”* Hyperedge formation fundamentally arises from intricate and non-uniformly distributed interactions among constituent nodes. For instance, in collaboration networks, core authors and peripheral participants exhibit distinct role disparities. Although mainstream approaches often employ heuristic aggregation strategies [8,29] using simplistic mean or average pooling operations, these methods fail to adequately address heterogeneous node influence. Specifically, they cannot capture the significant variations in contribution weights among nodes within the same hyperedge. Uniform aggregation operations inevitably dilute such critical semantic information. Moreover, order-specific hyperedge influence plays a pivotal role in node embedding formation, as hyperedges of varying scales (i.e., different orders) exhibit divergent semantic contributions to their participating nodes. However, existing methods lack explicit mechanisms to handle hyperedges of different orders, and the reliability of node–hyperedge influence mechanisms under varying order conditions remains largely unexplored.

***Challenge II: Hypergraph Augmentation.** “How can we generate a set of high-quality hypergraph-augmented views for CL while effectively capturing inter-view relationships?”* Hypergraph-augmented views are obtained by applying controlled perturbations to the original input data, thereby creating complementary representations for CL. The core challenge involves designing view generation rules that simultaneously preserve the topological structure and semantic integrity to ensure diverse yet informative perspectives, which can accurately capture both structural and attribute-based characteristics of hypergraphs. Existing approaches primarily rely on random augmentation strategies. For example, some methods construct augmented views by randomly dropping hyperedges [30], while others, such as TriCL [27], utilize random node feature masking and membership masking to enhance both attributes and structural information. Ref. [31] further categorizes four distinct hypergraph augmentation strategies. However, random perturbations may disrupt the intricate relational semantics within hyperedges and often neglect the inherent heterogeneity of group structures. Additionally, current methods predominantly perform contrastive learning by comparing node embeddings across different views, failing to incorporate hyperedge-level semantics and joint node–hyperedge relationships. Consequently, the learning process lacks sufficient guidance and constraints.

To effectively address the aforementioned challenges, we propose the Order propagation Fusion Self-supervised learning for Hyperedge prediction (OFSH) framework, featuring a Member-Aware Attention Aggregator (MHAA) that learns node importance weights and an Order-Specific Hyperedge Aggregator (OHAA) that groups neighborhood hyperedges by orders: it first extracts discriminative intra-group features via max–min pooling and then jointly models node influence and hyperedge order effects through inter-order attention propagation. Additionally, a key node-guided augmentation strategy with tri-level contrastive loss maximizes cross-view consistency at node, hyperedge, and association levels to enhance robustness against high-order structural perturbations. The main contributions are summarized as follows:

- We propose jointly modeling node influence heterogeneity and hyperedge order effects via order grouping with max–min pooling and design a dual-attention propagation architecture for dynamic node–hyperedge interaction modeling, significantly enhancing the semantic discriminability of embeddings.
- We propose a structure-aware view augmentation strategy guided by key nodes, combined with Adaptive Masking Probability Control, to generate diverse yet structurally faithful augmented views at both hyperedge and node levels. This effectively preserves the hypergraph’s core topology and semantics for robust contrastive learning.
- We construct collaborative optimization objectives at the node level, hyperedge level, and node–hyperedge interaction level, generating complementary self-supervised signals that more comprehensively model the multi-layered structural relationships in hypergraphs, effectively mitigating the data sparsity issue.
- Extensive experiments on five real-world hypergraph datasets demonstrate OFSH’s superiority and validate each module’s contribution, showing significant improvements over SOTA hyperedge prediction methods.

The remainder of this paper is organized as follows: Section 2 reviews recent research. Section 3 introduces preliminaries. Section 4 details our proposed methodology. We then conduct extensive experiments, comparing our approach with baseline methods, and present the results with analysis in Section 5. Finally, we conclude this paper in Section 6.

## 2. Related Work

### 2.1. Hyperedge Prediction Systems

Numerous studies have formulated the hyperedge prediction task as a classification problem. Expansion [7] progressively captures higher-order interaction information of different orders by stacking 2-to-n-order projected graphs (i.e., n-projected graphs) and employs logistic regression on the extracted features from these projected graphs to predict missing hyperedges. HyperSAGNN [29] adopts a self-attention-based graph neural network to process variable-sized hyperedges and computes the average probability of node groups forming hyperedges for prediction. NHP [10] first learns node embeddings via hyperedge-aware graph neural networks based on clique-expanded subgraphs and then aggregates the embeddings of nodes in target hyperedges using a max–min difference operation for prediction. The advanced method AHP [8] employs adversarial training to generate negative samples for model training and optimizes node aggregation through max–min pooling. CHESHIRE [32] utilizes hypergraph neural networks to learn node embeddings, enhances features via Chebyshev spectral graph convolutional layers, and combines max–min pooling with Frobenius norm pooling functions to aggregate node features within hyperedges for reaction prediction. CASH [25] leverages hypergraph neural networks to learn node embeddings and a context-aware node embedding aggregation mechanism, combining candidate node features in hyperedges for prediction.

However, these methods predominantly rely on node feature learning for hyperedge representation, generally ignoring the heterogeneity of node influence and limiting hypergraph structure exploration to shallow topological analysis. This paper further incorporates fine-grained considerations of node–hyperedge influence dynamics, aimed at improving prediction accuracy.

### 2.2. Hypergraph Attention Networks

To enhance information propagation efficiency between nodes and hyperedges in hypergraphs, numerous studies have focused on constructing hypergraph attention mechanisms [33,34]. Mainstream approaches typically either treat hyperedges as independent entities or transform them into fully connected subgraphs, directly transferring the Graph Attention Network (GAT) [35] framework for attention computation. Building upon this, Ref. [36] proposed a simplex attention method that separately implements attention mechanisms on simplices containing target high-order features and their associated low-order features. To further strengthen the expressive power of attention metrics, HyperGAT first introduced a serial two-level attention mechanism operating at both the node level and hyperedge level [20], while multi-scale attention in heterogeneous graph models focuses on learning feature aggregation weights for heterogeneous node or hyperedge types [37]. Additionally, some studies achieved attention modeling for neighbors at different distances through graph structure decomposition [38] or proposed various strategies to diversify attention sources and enhance representation capabilities [13].

However, these methods uniformly adopt a strategy of mixing hyperedges of different orders, failing to effectively distinguish and model the differentiated influences of order-specific hyperedges [39,40]. In reality, the order of hyperedges carries crucial semantic information, and explicitly differentiating and focusing on attention mechanisms for specific orders can significantly improve feature learning performance. To address this limitation, this paper proposes the combined MHAA and OHAA modules, aiming to achieve more precise and effective allocation of attention weights.

### 2.3. Hypergraph Self-Supervised Contrastive Learning

Recent studies have demonstrated that self-supervised learning can mitigate data sparsity issues in hypergraphs [41,42]. Representative work like HyperGene [43] employs dual-level (node-level and hyperedge-level) self-supervised tasks to model group relationships. However, its approach of converting hypergraphs into simple graphs via clique expansion leads to significant loss of higher-order information while failing to incorporate contrastive learning. Although HyperGCL [44] designs two view construction strategies—hyperedge drop (Drop Hyperedge) and membership masking (Membership Masking)—its random perturbation mechanism still compromises topological integrity. Ref. [42] alleviates data sparsity in group recommendation by proposing a dual node dropping strategy operating at both coarse and fine granularities. The state-of-the-art model TriCL [27] introduces a triple-way (node–group–member) contrastive mechanism; however, it has only been validated on node-level tasks without addressing hyperedge prediction scenarios. Moreover, TriCL adopts random hypergraph augmentation methods [31] while neglecting the inherent structural characteristics of original hypergraphs. CHGNN [26] implements adaptive hypergraph strategies to preserve primary topological structures, though its evaluation metrics for important nodes require more in-depth consideration.

To address these limitations, this paper proposes a key node-guided augmentation strategy that generates contrastive views while preserving higher-order semantic structures. Additionally, we design a triple-loss contrastive framework that significantly enhances performance specifically for hyperedge prediction tasks.

## 3. Preliminaries

In this section, we provide a concise overview of the essential definitions utilized throughout this paper.

**Definition** **1.** *Hypergraph: A hypergraph is denoted as G=(V,E), where $V=\{v_{1},v_{2},\ldots ,v_{\left|V\right|}\}$ is a node set and $E=\{e_{1},e_{2},\ldots ,e_{\left|E\right|}\}$ is a hyperedge set. It can be represented by the incident matrix H$\in {\left\{1,0\right\}}^{\left|V\right|\times \left|E\right|}$. Each hyperedge e∈E is a non-empty subset of V.*

**Definition** **2.** *Hypergraph Features: The node feature matrix of a hypergraph is denoted as $X\in R^{\left|V\right|\times d}$, where each row $x_{i}\in R^{d}$ of X is a d-dimension feature vector of the node $v_{i}$.*

**Definition** **3.** *Egonet of Nodes: We define the egonet of a node $v_{i}$ as the set of hyperedges that contains $v_{i}$; that is, $E_{\left\{v_{i}\right\}}=\;\left\{e_{j}\in E:v_{i}\in e_{j}\right\}$.*

**Definition** **4.** *Order of Hyperedges: We define the order of a hyperedge $e_{i}$ as the number of nodes in $e_{i}$; e.g., $e^{\langle 3\rangle }=\left\{v_{1},v_{2},v_{3}\right\}$ is a 3-order hyperedge. We define the maximum order of the egonet of node $v_{i}$ as $o_{v_{i}}^{\max}$. $E_{\left\{v_{i}\right\}}$ can further divided into l-1 sub-egonet sets $E_{\left\{v_{i}\right\}}=E_{\left\{v_{i}\right\}}^{\langle 2\rangle }\cup E_{\left\{v_{i}\right\}}^{\langle 3\rangle }\cup \cdots \cup E_{\left\{v_{i}\right\}}^{\langle l\rangle }$, where $E_{\left\{v_{i}\right\}}^{\langle l\rangle }$ is the l-order hyperedge sets of the node $v_{i}$.*

**Definition** **5.** *Set of Node Neighbors: We denote the set of all nodes belonging to at least one hyperedge containing the node $v_{i}$ as $N_{\left\{v_{i}\right\}}$.*

**Task** (Hyperedge Prediction Problem): Given a hypergraph G=(V,E), node feature matrix $X\in R^{\left|V\right|\times d}$, and incidence matrix $H\in {\left\{1,0\right\}}^{\left|V\right|\times \left|E\right|}$, the hyperedge prediction task is to determine whether a candidate hyperedge $e^{\prime }\in 2^{V}\setminus E$ is a valid hyperedge that may exist in the future or remain unobserved. In other words, the objective is to leverage the topological structure and feature information of the observed hypergraph to predict the formation of latent hyperedges not present in *E*.

## 4. Methodology

The hyperedge prediction process can be decomposed into three core components:

(1) Hypergraph Encoder: A hypergraph encoder, $f:\left(R^{\left|V\right|\times d},R^{\left|V\right|\times \left|E\right|}\right)\to \left(R^{\left|V\right|\times d},R^{\left|E\right|\times d}\right)$, generates both node and hyperedge embeddings from the observed hypergraph structure, i.e., f(X,H)=(P,Q).
(2) Hypergraph Node Aggregator: A node aggregator, $aggrt:\left(R^{\left|V\right|\times d},R^{\left|V\right|\times \left|E\right|}\right)\to R^{\left|V\right|\times d^{\prime }}$, employs various aggregation mechanisms to derive more precise node embeddings, which encapsulate rich topological structure information and node feature information.
(3) Hyperedge Candidate Scoring: After aggregating the node embeddings from $e^{\prime }$ to generate the final embedding for the hyperedge candidate, this representation is then fed into the predictor $pre:R^{d^{\prime }}\to R^{1}$ to compute the probability of the candidate hyperedge $e^{\prime }$ being formed.

**Figure 2:**
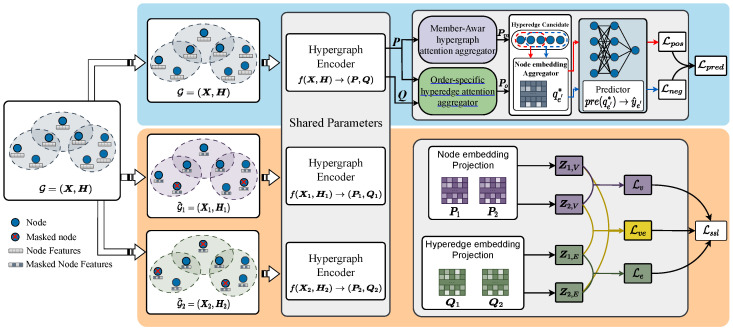
The overview framework of OFSH.

Based on this procedure, we jointly optimize the hypergraph encoder f(·), node aggregator aggrt(·), and hyperedge predictor pre(·) in an end-to-end manner by minimizing the loss L. Notably, the loss function L(·) is jointly defined by two tasks: hyperedge prediction as the primary task and self-supervised contrastive learning as the auxiliary task. The overall framework of OFSH is illustrated in Figure 2. The details of f(·), aggrt(·), pre(·), and L(·) are elaborated in Section 4.2, Section 4.3, and Section 4.4, respectively.

### 4.1. Hypergraph Encoder

The hypergraph encoder employs a bidirectional message-passing mechanism to capture higher-order relationships inherent in hypergraphs. Unlike graphs that only model pairwise relations, hyperedges connect multiple nodes, representing group interactions. This design is motivated by the prior work [29], which shows that aggregating information from nodes to hyperedges and vice versa allows the model to learn embeddings that encapsulate the complex semantics of hyperedges, specifically the following:

(1) Node to hyperedge: Aggregates features of nodes within a hyperedge to form a hyperedge embedding, capturing the collective properties of the group.
(2) Hyperedge to node: Propagates hyperedge features to nodes, enabling each node to integrate context from all hyperedges it belongs to, thus reflecting its participation in higher-order structures.

Based on this, OFSH updates the embedding of each hyperedge by aggregating the features of its constituent nodes and subsequently updates each node’s embedding based on the features of its affiliated hyperedges. Formally, given the hypergraph incidence matrix H and initial node features X, the embeddings of nodes and hyperedges in the l-th layer can be defined as follows:

(1) $$\begin{array}{cc} \; & q_{e_{i}}^{\left(l\right)}=\sigma \left(\sum_{v_{j}\in e_{i}}\frac{p_{v_{j}}^{\left(l-1\right)}W_{E}^{\left(l\right)}}{\left|e_{i}\right|}+b_{E}^{\left(l\right)}\right)\\ \; & p_{v_{i}}^{\left(l\right)}=\sigma \left(\sum_{e_{j}\in E_{\left\{v_{i}\right\}}}\frac{q_{e_{j}}^{\left(l\right)}W_{V}^{\left(l\right)}}{\left|E_{\left\{v_{i}\right\}}\right|}+b_{V}^{\left(l\right)}\right) \end{array}$$

where $W_{*}^{\left(k\right)}\in R^{d\times d}$ denotes a learnable matrix, $p_{v_{i}}^{\left(0\right)}=x_{i}$, and $x_{i}$ is the initial feature vector of node $v_{i}$. $q_{e_{i}}^{\left(l\right)}$ and $p_{v_{i}}^{\left(l\right)}$ denote the embeddings of edge $e_{i}$ and node $v_{i}$ at the l-th iteration. As shown in Figure 2, the learnable parameters in the hypergraph encoder are shared in the self-supervised part. Equation (1) can be rewritten in the following form of Equation (2), and the hyperedge embeddings $Q^{\left(l\right)}=\left[q_{e_{i}}^{\left(l\right)}\right]\in R^{|E|\times d}$ and node embeddings $P^{\left(l\right)}=\left[p_{v_{i}}^{\left(l\right)}\right]\in R^{|V|\times d}$ at the l-th layer are defined as follows:

(2) $$\begin{array}{cc} \; & Q^{\left(l\right)}=\sigma \left({\left(D^{E}\right)}^{-1}H^{T}P^{\left(l-1\right)}W_{E}^{\left(l\right)}+b_{E}^{\left(l\right)}\right)\\ \; & P^{\left(l\right)}=\sigma \left({\left(D^{V}\right)}^{-1}H\;Q^{\left(l\right)}W_{V}^{\left(l\right)}+b_{V}^{\left(l\right)}\right) \end{array}$$

where $P^{\left(0\right)}=\sigma \left(X\cdot W_{1}\right)\in R^{|V|\times d}$. $W_{*}^{\left(k\right)}\in R^{d\times d}$ and $b_{*}^{\left(k\right)}\in R^{d}$ are the trainable weight and bias matrices, respectively. σ denotes a nonlinear activation function. And $d_{ii}^{E}\in D^{E}$ represents the number of non-zero elements in the *i*-th row of matrix $H^{T}$, while $d_{ii}^{IV}\in D^{V}$ is the number of non-zero elements in the *i*-th row of matrix H.

Finally, the hypergraph encoder module outputs the hyperedge features $Q\in R^{|E|\times d}$ and node features $P\in R^{|V|\times d}$. These embeddings are used as input to the hypergraph attention aggregator (Section 4.2) to compute attention weights and refine the embeddings further.

### 4.2. Hypergraph Attention Aggregator

The attention mechanism serves a pivotal role in hypergraph node aggregation by dynamically computing relationship strengths between nodes and hyperedges. This learnable weighting scheme provides an adaptive mechanism for information aggregation, enabling more accurate modeling of complex dependencies and structural patterns in hypergraphs while overcoming the limitations of simplistic averaging approaches. Inspired by the SAN [36] framework, we propose a novel Member-Aware Hypergraph Attention Aggregator (MHAA) that explicitly captures the individual contributions of member nodes within hyperedges. This addresses the inherent bias in conventional methods that depend exclusively on degree information, thereby significantly enhancing the expressive power of node embeddings. Moreover, to account for the influence of hyperedge order heterogeneity on node betweenness distribution, we introduce an Order-Specific Hyperedge Attention Aggregator (OHAA). This component computes order-specific attention weights, enabling the model to effectively adapt to diverse higher-order structures and improving the discriminative capability of node embeddings in complex interaction scenarios. By integrating these two complementary attention mechanisms, our approach augments feature learning from multiple perspectives, consequently improving the model’s performance in hyperedge prediction tasks.

#### 4.2.1. Member-Aware Hypergraph Attention Aggregator (MHAA)

Existing heuristic aggregation methods (e.g., mean pooling) indiscriminately assign equal weights to all nodes within a hyperedge, completely neglecting the substantial variations in their functional roles (e.g., director versus supporting actor and lead author versus contributing author). This limitation inevitably leads to hyperedge representations that cannot effectively discriminate between meaningful semantic patterns (e.g., distinct movie genres or varying paper quality tiers). In marked contrast, our proposed MHAA framework incorporates a trainable attention mechanism to dynamically determine the relative importance (attention weights) of each node when aggregating their embeddings to construct hyperedge representations. This sophisticated approach allows the model to discern the differential roles and contributions of individual nodes during hyperedge prediction, thus more precisely capturing the intrinsic dynamics of hyperedge formation. For instance, the mechanism can effectively recognize the dominant influence of director nodes and accordingly assign them higher weights. Consequently, the node-to-hyperedge importance scores and the member-aware embeddings for a hyperedge can be formally expressed as follows:

(3) $$\alpha_{e_{i},v_{j}}=\frac{\exp\left(\sigma (W_{m}q_{m,e_{i}}∥W_{m}p_{m,v_{j}})\right)}{\sum_{v_{h}\in e_{i}}\exp\left(\sigma (W_{m}q_{m,e_{i}}∥W_{m}p_{v_{h}})\right)},v_{j}\in e_{i}$$

(4) $$q_{m,e_{i}}=\sum_{v_{j}\in e_{i}}\alpha_{e_{i},v_{j}}\cdot p_{v_{j}}$$

where $\alpha_{e_{i}}$ denotes the attention score of node $v_{j}$ in edge $e_{i}$ during the *l*-th iteration and $p_{v_{j}}$ represents the node embedding obtained after hypergraph feature encoding.

The hyperedge vectors aggregated through attention mechanisms are mapped to the node vectors, yielding

(5) $$p_{m,v_{i}}=p_{v_{i}}+\sum_{e_{j}\in E_{\left\{v_{i}\right\}}}\frac{q_{m,e_{j}}}{\left|E_{\left\{v_{i}\right\}}\right|}$$

Finally, we derive hyperedge embeddings $Q_{m}$ and node embeddings $P_{m}$ through the MHAA mechanism for subsequent hypergraph prediction tasks.

#### 4.2.2. Order-Specific Hyperedge Attention Aggregator (OHAA)

**Figure 3:**
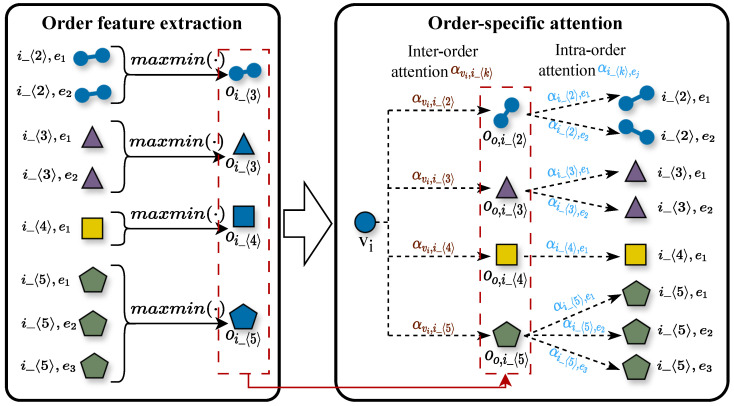
Order-Specific Hyperedge Attention Aggregator.

Nodes frequently participate in hyperedges of diverse orders (e.g., 2-order social relationships, 5-order paper collaborations, and 10-order community activities). However, most existing methods either ignore or homogenize the distinct and non-equivalent influences of these hyperedges on node representation. Specifically, current approaches either disregard hyperedge order information or apply uniform aggregation (analogous to equal weighting) indiscriminately across all hyperedge orders, leading to insufficient discriminative power in node embeddings and the loss of critical node contextual information. To systematically address this limitation, we propose the Order-aware Hyperedge Aggregation with Attention (OHAA) module, inspired by hierarchical networks [45]. The OHAA module organizes node neighborhood hyperedges into order-specific groups. Within each group, we employ max–min pooling [10] to enhance feature discriminability while preventing the dominance of highly active hyperedges, thereby generating order-specific embeddings. Subsequently, an attention mechanism learns differential weights for node features across different order groups, producing comprehensive order-aware node embeddings that accurately reflect both the importance of multi-scale relationships and the role-specific characteristics of nodes. This design enables the OHAA module to not only capture the differential impacts of hyperedges across orders but also facilitate the propagation of higher-order information in the global semantic space, significantly improving the model’s expressive power. Figure 3 illustrates the architecture of the OHAA module.

Unlike traditional sum or average pooling, max–min pooling explicitly computes the range of feature dimensions to capture the feature dispersion within same-order hyperedge sets. In hypergraph scenarios, hyperedges of the same order share identical sizes but may exhibit significant heterogeneity in their internal node interaction patterns (e.g., some hyperedges have compact structures while others are sparse). Sum or mean operations obscure such structural differences due to smoothing effects:

1. Sum pooling amplifies high-frequency features (e.g., highly active nodes) but neglects divergence in feature distributions.
2. Average pooling compresses group features toward the centroid, causing the loss of discriminative signals across hyperedges.
3. Max–min pooling preserves two critical types of information: (1) extraction of the strongest, most representative signals (e.g., core node features) and (2) capture of anomalies or boundary patterns within the group. The range between these directly quantifies intra-group heterogeneity. This property makes max–min pooling particularly suited for modeling topological relationships that share the same order but are structurally heterogeneous, thereby enhancing embedding discriminability. Subsequent experiments (Section 5.5.3) validate the superiority of this mechanism for groups of hyperedges of the same order.

Generally, nodes within a hyperedge can consist of vastly different or complementary elements (e.g., acids and bases in chemical reactions) or conversely be composed of a sequence of similar nodes. To better accommodate these two distinct compositional patterns of hyperedges, we employ a max–min optimization approach to extract homogeneous-order hyperedge information:

(6) $$o_{i\_\langle k\rangle }:=\sigma \left(W\cdot \mathrm{maxmin}\left\{q_{e_{j}}:e_{j}\in E_{\left\{v_{i}\right\}}^{\langle k\rangle }\right\}\right)$$

where $o_{i\_\langle k\rangle }$ represents the vector of hyperedge features of *k* order of node $v_{i}$ and $q_{e_{j}}$ is the feature embedding of the hyperedge $e_{j}$ after hypergraph feature encoding. Given a hyperedge $e_{i}=\left\{v_{1},,v_{2}\cdots v_{j}\right\}$, the function

(7) $$\mathrm{maxmin}\left\{q_{e_{j}}:e_{j}\in E_{\left\{v_{i}\right\}}^{\langle k\rangle }\right\}={\left(\max_{e_{s}\in E_{\left\{v_{i}\right\}}^{\langle k\rangle }}Q_{sl}-\min_{e_{t}\in E_{\left\{v_{i}\right\}}^{\langle k\rangle }}Q_{tl}\right)}_{l=1,\cdots ,d}$$

represents the element-wise differential between the maximum and minimum values of hyperedge feature vectors. This max–min differential operator enables simultaneous capture of hyperedge structural information from nodes with similar features while quantifying connections between nodes exhibiting significantly divergent characteristics. Subsequently, these ordered hyperedge features are processed by the feature aggregation module for attention computation and representation learning.

Subsequently, we compute the node-to-order attention and order-to-hyperedge attention, respectively:

(8) $$\alpha_{i\_\langle k\rangle ,e_{j}}=\frac{\exp\left(\sigma (W_{o1}o_{i\_\langle k\rangle }∥W_{o1}q_{e_{j}})\right)}{\sum_{h=2}^{E_{\left\{v_{i}\right\}}^{\langle k\rangle }}\left(\sigma (W_{o1}p_{v_{i}}∥W_{o1}q_{e_{j}})\right)},e_{j}\in E_{\left\{v_{i}\right\}}^{\langle k\rangle }$$

(9) $$\alpha_{v_{i},i\_\langle k\rangle }=\frac{\exp\left(\sigma (W_{o2}p_{v_{i}}∥W_{o2}o_{i\_\langle k\rangle })\right)}{\sum_{j=2}^{o_{v_{i}}^{\max}}\left(\sigma (W_{o2}p_{v_{i}}∥W_{o2}o_{i\_\langle j\rangle })\right)},2\leq k\leq o_{v_{i}}^{\max}$$

where $\alpha_{i\_\langle k\rangle ,e_{j}}$ is the attention to the hyperedge embedding of $e_{j}$ relative to the *k*-order hyperedge embedding $o_{i\_\langle k\rangle }$, while $\alpha_{v_{i},i\_\langle k\rangle }$ is the attention to the embedding of the *k*-order hyperedge $o_{i\_\langle k\rangle }$ relative to the node $v_{i}$. $o_{v_{i}}^{\max}$ is the maximum order of the egonet of node $v_{i}$ and $W_{o1}$ and $W_{o2}$ are trainable parameters of OHAA.

Subsequently, we employ order propagation as an intermediate bridge to aggregate hyperedge features, thereby forming node feature embeddings.

(10) $$o_{o,i\_\langle k\rangle }=\sum_{e_{j}\in E_{\left\{v_{i}\right\}}}\alpha_{i\_\langle k\rangle ,e_{j}}\cdot q_{e_{j}}$$

(11) $$p_{o,v_{i}}=p_{v_{i}}+\sum_{e_{j}\in E_{\left\{v_{i}\right\}}}\alpha_{v_{i},i\_\langle k\rangle }\cdot o_{o,i\_\langle k\rangle }$$

where $o_{o,i\_\langle k\rangle }$ serves as the k-order hyperedge embedding that functions as the intermediate bridge.

Finally, we obtain hyperedge embeddings $Q_{o}$ and node embeddings $P_{o}$ through MHAA, which will be employed for subsequent hypergraph prediction tasks.

### 4.3. Hyperedge Prediction

After obtaining the node embeddings $P_{m}$ and $P_{o}$ generated by the MHAA and OHAA modules, respectively, we formulate the hyperedge prediction task as a two-stage pipeline: (1) fusing the node features from both views to obtain a unified hyperedge representation and (2) predicting the existence probability through a multi-layer perceptron. The supervised loss $L_{pred}$, together with the self-supervised contrastive loss described in Section 4.4, is jointly optimized during training. During inference, the learned node representations are directly employed for prediction without additional optimization.

First, we fuse node features from two attention perspectives, with the final node features being

(12) $$P^{*}=\frac{1}{2}\left(P_{m}+P_{o}\right)$$

Next, given a hyperedge candidate $e^{\prime }=\left\{v_{1}^{\prime },v_{2}^{\prime },\cdots ,v_{\left|e^{\prime }\right|}^{\prime }\right\}$, we aggregate node embeddings based on their respective influence within the hyperedge structure:

(13) $$\begin{array}{cc} \; & q_{e^{\prime }}^{*}=\sum_{v_{i}^{\prime }\in e^{\prime }}\alpha_{v_{i}^{\prime }}\cdot p_{v_{i}^{\prime }}^{*}W_{f,agg}\\ \; & \alpha_{v_{i}^{\prime }}=\frac{\exp\left(\sigma (W_{f}p_{v_{i}^{\prime }}^{*})\right)}{\sum_{v_{j}^{\prime }\in e^{\prime }}\exp\left(\sigma (W_{f}p_{v_{j}^{\prime }}^{*})\right)},v_{i}^{\prime }\in e^{\prime } \end{array}$$

Ultimately, hyperedge embbedings are processed through a multi-layer perceptron to derive the probabilistic likelihood of hyperedge formation:

(14) $${\hat{y}}_{e^{\prime }}=f_{pred}\left(q_{e^{\prime }}^{*}\right)=sigmoid(W_{mlp}q_{e^{\prime }}^{*})$$

*Model Training*: For model training and validation, we consider both positive and negative examples (i.e., existing and non-existent hyperedges). Specifically, we employ the following heuristic-based negative sampling method [28] to generate negative instances:

- Sized Negative Sampling (SNS): Randomly selecting k nodes without considering the network structure.
- Clique NS (CNS): Choosing a hyperedge and substituting one of its nodes with another that is adjacent to all remaining nodes in the hyperedge.
- Motif NS (MNS): Sampling a k-connected component in a clique-expanded hypergraph.

The optimization objective is to train the model parameters such that positive samples attain higher prediction scores while negative samples obtain lower values. Specifically, let $E^{\prime }$ denote the set of potential hyperedges; the prediction loss function is formulated as follows:

(15) $$L_{pred}=-\frac{1}{\left|E^{\prime }\right|}\sum_{e^{\prime }\in E^{\prime }}\left[y_{e^{\prime }}log\left({\hat{y}}_{e^{\prime }}\right)+\left(1-y_{e}\right)log\left(1-{\hat{y}}_{e^{\prime }}\right)\right]$$

where $L_{pred}$ denotes the loss value of supervised training, encompassing both positive and negative samples. $y_{e^{\prime }}=1$ indicates that the hyperedge candidate is a positive hyperedge, whereas $y_{e^{\prime }}=0$ denotes a negative hyperedge candidate.

### 4.4. Hypergraph Augmentation

The hypergraph augmentation generates multi-view augmented representations by introducing controlled perturbations to the original structure, enabling the model to capture stable latent semantic features through contrastive learning. Inspired by GraphCL [31] and GCA [46], we propose an importance-adaptive view generation scheme to maximally preserve the core topology and critical information: the approach dynamically adjusts node preservation probabilities based on importance metrics, preferentially maintaining crucial nodes and hyperedges during perturbation to ensure semantic integrity of augmented views. By strategically preserving vital topological and semantic information, this scheme substantially enhances contrastive learning performance for classification tasks while mitigating the risk of semantic structure damage caused by random perturbations.

#### 4.4.1. Hypergraph Key Node Identification

Contrastive learning methods aim to learn representations that remain invariant to data augmentations while maximizing inter-view consistency. However, conventional random augmentation approaches exhibit significant limitations—their indiscriminate dropping or masking of hyperedges/features may disrupt high-order semantic structures. For instance, completely masking all members within a hyperedge effectively severs group relationships, which ultimately undermines the effectiveness of contrastive learning.

To address these limitations, we propose a novel strategy for generating augmented views that preserves essential topological structures. Our approach consists of two key steps: First, we identify pivotal nodes, which contribute most significantly to maintaining structural stability in hypergraphs. Preserving these connectivity hubs ensures that perturbations primarily affect non-critical regions, thereby retaining the original semantic skeleton while simultaneously providing diversified perspectives. This strategy not only enables the model to fully exploit the underlying semantics of the hypergraph but also establishes a solid foundation for adaptive view generation.

- Local Influence Evaluation

When assessing a node’s local influence, it is essential to comprehensively evaluate its topological connectivity from dual perspectives: Firstly, the number of neighbors directly reflects the breadth of connections—a higher neighbor count indicates stronger connectivity. Secondly, fewer hyperedges shared with neighbors (indicating lower overlap) suggests that information pathways rely more heavily on this node, thereby highlighting its pivotal role in maintaining the structural integrity of the hypergraph.

**Figure 4:**
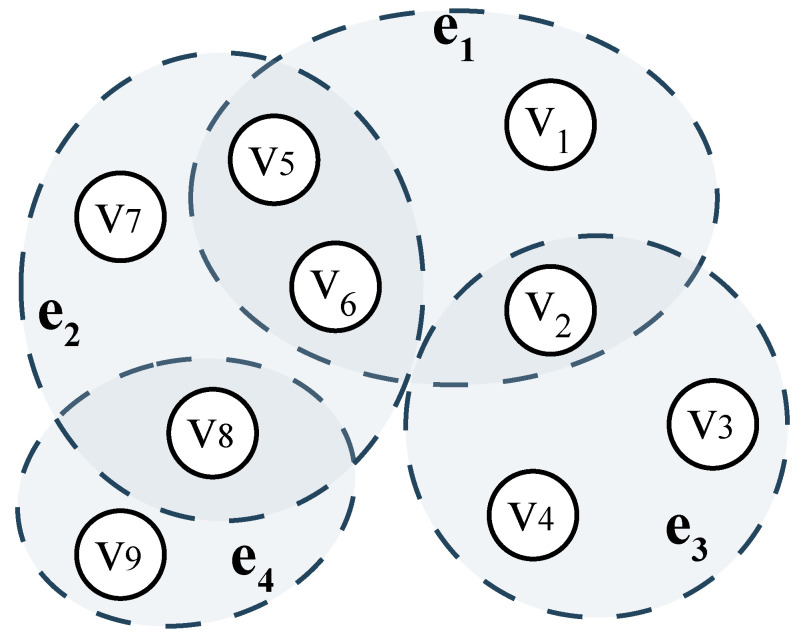
A hypergraph model for illustration.

**Example** **1.** *In Figure 4, $E_{\left\{v_{2}\right\}}=\left\{e_{1},e_{3}\right\}$ contains six nodes, $v_{i}\left(1\leq i\leq 6\right)$, while $E_{\left\{v_{8}\right\}}=\left\{e_{2},e_{4}\right\}$ consists of 5 nodes: $v_{i}\left(5\leq i\leq 9\right)$. Under these circumstances, removing node $v_{2}$ would cause greater connectivity loss to the hypergraph than removing $v_{8}$. Furthermore, $E_{\left\{v_{2}\right\}}$ and $E_{\left\{v_{6}\right\}}$ contain an identical number of nodes (6 each). However, $v_{6}$ is structurally less critical for two reasons:* ***(i)*** *$E_{\left\{v_{6}\right\}}⋂E_{\left\{v_{5}\right\}}=\left\{e_{1},e_{2}\right\}$. This means that the destruction of $v_{6}$ would not isolate $e_{1}$ and $e_{2}$, as they remain connected via $v_{5}$.* ***(ii)*** *$N_{\left\{v_{3}\right\}}⋂N_{\left\{v_{1}\right\}}=v_{2}$ and $N_{\left\{v_{4}\right\}}⋂N_{\left\{v_{1}\right\}}=v_{2}$. The failure of $v_{2}$ would disconnect $e_{1}$ and $e_{3}$, since no alternative paths exist between these hyperedges.*

Based on the aforementioned principles and examples, this paper defines the connectivity of nodes in a hypergraph to quantify their pivotal role in information propagation.

**Definition** **6.** *Connectivity of nodes in a hypergraph:*

(16) $$c\left(v_{i}\right):=\frac{\left|{⋃}_{e\in E_{\left\{v_{i}\right\}}}e\right|}{\frac{1}{\left|N_{\left\{v_{i}\right\}}\right|}\sum_{v_{j}\in N_{\left\{v_{i}\right\}}}\left|E_{\left\{v_{j}\right\}}⋂E_{\left\{v_{i}\right\}}\right|}$$

The connectivity value decreases when the number of neighbor nodes increases while the number of shared hyperedges with neighbors decreases, indicating higher node importance.

To verify whether the aforementioned $c(v_{i})$ truly reflects node $v_{i}$’s capability to perform hub-like information transmission, we take the graph in Figure 4 as an example and compute the connectivity for four representative nodes. The calculated values show that $c(v_{1})=4$, $c(v_{2})=6$, $c(v_{6})=5$, and $c(v_{9})=2$, so we can conclude that $c\left(v_{9}\right)<c\left(v_{1}\right)<c\left(v_{6}\right)<c\left(v_{2}\right)$, which is also consistent with our intuitive judgment.

Alternatively, we normalize the computed connectivity values of all nodes to obtain their local relative importance.

- Global Influence Evaluation

The global influence of nodes should account for the homogeneity of group relationships in hypergraphs. Inspired by the concept of hyperedge homogeneity [47], we propose the following.

**Definition** **7.** *The homophily of intra-group nodes:*

(17) $$\mathrm{homo}\left(v_{i},v_{j}\right):=\frac{\left|E_{\left\{v_{i},v_{j}\right\}}\right|}{\left|E_{\left\{v_{i}\right\}}⋃E_{\left\{v_{j}\right\}}\right|}$$

$\mathrm{homo}(v_{i},v_{j})$ represents the semantic similarity between nodes $v_{i}$ and $v_{j}$ in the hypergraph structure.

In the PageRank algorithm, nodes traverse to their adjacent nodes with equal probability. However, in real-world hypergraph networks, nodes do not uniformly distribute their transitions among neighbors—they consistently exhibit a preference for navigating toward more homogeneous nodes. To address this, we propose an enhanced PageRank algorithm to quantify the global importance of nodes. During the initialization phase, the PageRank (PR) values are distributed to neighboring nodes based on the intra-group hyperedge homogeneity. This process iterates until the pr values of all nodes in the hypergraph network converge to a stable state:

(18) $$pr\left(v_{i}\right)=\frac{1-\lambda }{\left|V\right|}+\lambda \;\sum_{e_{j}\in E_{\left\{v_{i}\right\}}}\sum_{v_{k}\in e_{j}}\mathrm{homo}\left(v_{i},v_{k}\right)\cdot pr\left(v_{k}\right)$$

where $pr(v_{i})$ denotes the continuously updated global importance score of node $v_{i}$ and λ represents the damping factor.

Subsequently, we integrate global influence and local influence to comprehensively evaluate a node’s structural backbone role in the network, formally defined as follows:

(19) $$w_{v_{i}}=\alpha \cdot pr\left(v_{i}\right)+\left(1-\alpha \right)\cdot c\left(v_{i}\right)$$

where α is a balancing factor that determines the weighting ratio between local influence and global influence. $w_{v_{i}}$ quantitatively integrates a node’s role as a topological backbone, providing a basis for adaptive augmentation view generation.

#### 4.4.2. Adaptive Hypergraph View Construction

In adaptive hypergraph view construction, we adjust the masking probabilities differentially based on node importance to preserve both the topological skeleton and high-order semantic structural invariance. Inspired by findings demonstrating the enhanced benefits of combining different augmentations [31], we jointly employ two heterogeneous strategies: hyperedge-wise topological augmentation and node-wise feature augmentation. By generating diverse perturbation patterns, this approach effectively alleviates the model’s over-reliance on local low-level features, consequently improving both feature generalizability and the robustness of core semantic representations.

- Hyperedge-wise Topology Augmentation.

For topological-level augmentation, we randomly mask nodes within hyperedges according to a specific probability. Formally, for each hyperedge $e_{i}$, we sample a modified hyperedge subset ${\tilde{e}}_{i}$ from $e_{i}$ to the following node masking probability:

(20) $$P\left(v_{j}\in {\tilde{e}}_{i}\right)=1-p_{v_{j}}^{e_{i}}$$

where $p_{v_{j}}^{e_{i}}$ is the masking probability of node $v_{j}$ in hyperedge $e_{i}$ and ${\tilde{e}}_{i}$ is used as the hyperedge set in view generation. The probability $p_{v_{j}}^{e_{i}}$ should quantify the importance of node $v_{j}$ in the hyperedge, as this augmentation strategy preferentially preserves more critical topological structures while disrupting less important edges with higher probability.

Next, the masking probability is calculated based on the node comprehensive influence scores obtained from Section 4.4.1. To address the potential large variation in comprehensive influence values among important nodes [48], we initially set $s_{v_{i}}=\log w_{v_{i}}$ to prevent high-density nodes from excessively dominating the results. Subsequently, these adjusted values are normalized to form a probability distribution:

(21) $$p_{v_{i}}=\min\left(\frac{s_{v_{i},\max}-s_{v_{i}}}{s_{v_{i},\max}-\mu_{v}}\cdot p_{node},p_{\tau }\right)$$

where $p_{node}$ is a hyperparameter controlling the overall probability of masking any node and $s_{v_{i},\max}$ and $\mu_{v}$ represent the maximum and average values in $s_{v_{i}}$, respectively. $p_{\tau }<1$ serves as a truncation probability to prevent excessive disruption of the hypergraph topology.

- Node-wise Feature Augmentation.

For feature-level augmentation, we employ noise injection through dimension-wise masking of node features. Formally, given a feature dimension $d_{i}$, we generate a modified mask vector $\tilde{m}={\left\{0,1\right\}}^{\left|D\right|}$ by sampling from the following probability distribution:

(22) $$P\left(d_{i}\in D\right)=1-p_{d_{i}}$$

Then, the masked node feature $\tilde{X}$ is computed by

(23) $$[\tilde{X}={\left[{\tilde{m}}_{1}\circ x_{1};{\tilde{m}}_{2}\circ x_{2};\cdots ;{\tilde{m}}_{n}\circ x_{n}\right]}^{T}$$

Similarly to topological-level augmentation, the probability parameter $p_{d_{i}}$ should reflect the importance of the *i*-th feature dimension. We hypothesize that feature dimensions frequently appearing in influential nodes should be considered more significant. Accordingly, we employ node importance as a proxy to quantify feature dimension importance:

(24) $$w_{d_{i}}=\sum_{v_{j}\in V}\left|x_{j,d_{i}}\right|\cdot w_{v_{j}}$$

where $w_{v_{j}}$ represents the comprehensive influence of node $v_{j}$, and $x_{j,d_{i}}$ denotes the one-hot value of the $d_{i}$-dimensional feature of node $v_{j}$. $w_{d_{i}}$ indicates the aggregated influence of the $d_{i}$-dimension node feature across all feature dimensions. For example, in citation networks, keywords (i.e., node features along specific dimensions) pertaining to highly cited papers should naturally be recognized as more significant and influential.

Subsequently, we normalize the weights to obtain probabilities that quantify the importance of node features. Formally,

(25) $$p_{f_{i}}=\min\left(\frac{s_{d_{i},\max}-s_{d_{i}}}{s_{d_{i},\max}-\mu_{d}}\cdot p_{f},p_{\tau }\right)$$

where $p_{f}$ is a hyperparameter that controls the global probability of masking any dimension of the features. $s_{d_{i}}=\log w_{d_{i}}$, while $s_{d_{i},\max}$ and $\mu_{d}$ represent the maximum and mean values in $s_{d_{i}}$, respectively.

By jointly performing topological-level and feature-level augmentations, we generate two masked views, denoted as ${\tilde{G}}_{1}=\left(X_{1},H_{1}\right)$ and ${\tilde{G}}_{2}=\left(X_{2},H_{2}\right)$.

#### 4.4.3. Self-Supervised Hypergraph Contrastive Learning

For the two augmented views ${\tilde{G}}_{1}=\left(X_{1},H_{1}\right)$ and ${\tilde{G}}_{2}=\left(X_{2},H_{2}\right)$ generated by the adaptive augmentation strategy driven by key nodes, we employ a shared-parameter hypergraph encoder consistent with Section 4.1’s backbone task to derive node embeddings $P_{k}$ and hyperedge embeddings $Q_{k}$ (where k=1,2 corresponds to the two augmentation views). Subsequently, we introduce two projection heads $g_{V}\left(\cdot \right):R^{\left|V\right|\times d}\to R^{\left|V\right|\times d}$ and $g_{E}\left(\cdot \right):R^{\left|V\right|\times d}\to R^{\left|V\right|\times d}$ to refine the representations used for constructing contrastive loss. Specifically, given the learned node embedding $P_{k}$ and hyperedge embedding $Q_{k}$ from the *k*-th augmented view, their projected embeddings $Z_{k,V}$ and $Z_{k,E}$ are defined as follows:

(26) $$Z_{k,V}=g_{V}\left(P_{k}\right),\;Z_{k,E}=g_{E}\left(Q_{k}\right),\;$$

where $g_{*}\left(\cdot \right)$ employs a two-layer MLP with ELU nonlinear activation functions for projection. This projection operation helps alleviate information bottlenecks and enhances the quality of representations in contrastive learning.

Based on the projected node embeddings $Z_{k,V}$ and hyperedge embeddings $Z_{k,E}$, we measure and minimize the discrepancies between the two views across three complementary levels (i.e., the triple contrastive loss) to more comprehensively capture structural information in the hypergraph, thereby improving hyperedge prediction performance. These discrepancy signals collectively provide rich self-supervised information to complement the main task.

- Node–Node Contrast Loss.

This loss function aims to maximize the embedding similarity of the same node across different views while pushing apart the embeddings of different nodes. For a given node $v_{i}$, its embedding $z_{1,v_{i}}$ from view ${\tilde{G}}_{1}$ and embedding $z_{2,v_{i}}$ from view ${\tilde{G}}_{2}$ form a positive sample pair, while its embeddings paired with cross-view embeddings of other nodes constitute negative sample pairs. We employ the normalized temperature-scaled cross-entropy loss [49], formulated as follows:

(27) $$L_{v}=\frac{1}{2\left|V\right|}\sum_{v_{i}\in V}\left(\ell_{n}\left(z_{1,v_{i}},z_{2,v_{i}}\right)+\ell_{n}\left(z_{2,v_{i}},z_{1,v_{i}}\right)\right)$$

where $\ell_{n}$ is defined as follows:

(28) $$\ell_{n}\left(z_{1,v_{i}},z_{2,v_{i}}\right)=-\log\frac{\exp\left(s\left(z_{v,v_{i}}^{1},z_{v,v_{i}}^{2}\right)/\tau_{v}\right)}{\sum_{v_{i}\in V}\exp\left(s\left(z_{v,v_{i}}^{1},z_{v,v_{j}}^{2}\right)/\tau_{v}\right)}$$

where s(·) is the cosine similarity operation and $\tau_{v}$ is a temperature parameter corresponding to $\ell_{v}$.

- Hyperedge–Hyperedge Contrast Loss.

For a hyperedge, its hyperedge embedding $z_{1,e_{i}}$ from view ${\tilde{G}}_{1}$ and $z_{2,e_{i}}$ from view ${\tilde{G}}_{2}$ form a positive sample pair, while the embeddings of this hyperedge with other cross-view hyperedges constitute negative sample pairs. The form of the loss function is similar to that at the node–node level:

(29) $$L_{e}=\frac{1}{2\left|E\right|}\sum_{e_{i}\in E}\left(\ell_{e}\left(z_{1,e_{i}},z_{2,e_{i}}\right)+\ell_{e}\left(z_{2,e_{i}},z_{1,e_{i}}\right)\right)$$

(30) $$\ell_{e}\left(z_{1,e_{i}},z_{2,e_{i}}\right)=-\log\frac{\exp\left(s\left(z_{e,e_{i}}^{1},z_{e,e_{i}}^{2}\right)/\tau_{e}\right)}{\sum_{e_{j}\in E}\exp\left(s\left(z_{e,e_{i}}^{1},z_{e,e_{j}}^{2}\right)/\tau_{e}\right)}$$

where $\tau_{e}$ is a temperature parameter corresponding to $\ell_{e}$.

- Node–Hyperedge Contrast Loss.

This loss function aims to enhance the invariance of the relationship between anchor nodes and their associated crossing hyperedges across different views. Consider the assumptions that $\tilde{E}=\left\{e_{1},e_{2},e_{3}\right\}$ and ${\tilde{E}}_{\left\{v_{i}\right\}}=\left\{e_{1},e_{2}\right\}$, where $v_{i}$ serves as an anchor node. Then $z_{1,e_{1}}$ and $z_{2,e_{1}}$ constitute positive samples of each other (with $z_{2,v_{1}}$ and $z_{1,e_{2}}$ being mutually positive as well), while $z_{1,v_{1}}$ and $z_{2,e_{3}}$ form negative samples. The node–hyperedge contrast loss is formally defined as follows:

(31) $$L_{ve}=\frac{1}{2M}\sum_{v_{i}\in V}\sum_{e_{j}\in E_{\left\{v_{i}\right\}}}\left(\ell_{ne}\left(z_{1,v_{i}},z_{2,e_{j}}\right)+\ell_{ne}\left(z_{2,v_{i}},z_{1,e_{j}}\right)\right)$$

where $M=\sum_{v_{i}\in V}\sum_{e_{j}\in E_{\left\{v_{i}\right\}}}\left|e_{j}\right|$ and $\ell_{ve}$ is defined as follows:

(32) $$\ell_{ve}\left(z_{1,v_{i}},z_{2,e_{j}}\right)=-\log\frac{\exp\left(D\left(z_{1,v_{i}},z_{2,e_{j}}\right)/\tau_{ve}\right)}{\sum_{v^{\prime }\in V}\exp\left(D\left(z_{1,v^{\prime }},z_{2,e_{j}}\right)/\tau_{ve}\right)}-\log\frac{\exp\left(D\left(z_{1,v_{i}},z_{2,e_{j}}\right)/\tau_{ve}\right)}{\sum_{e^{\prime }\in E}\exp\left(D\left(z_{1,v^{\prime }},z_{2,e^{\prime }}\right)/\tau_{ve}\right)}$$

where $D\left(x,y\right)$ is a discriminator that represents the probability scores assigned to this node–hyperedge representation pair. $\tau_{ve}$ is a temperature parameter corresponding to $\ell_{ve}$.

The three complementary loss functions are combined by summation to yield the total self-supervised contrastive learning loss:

(33) $$L_{ssl}=L_{v}+L_{e}+L_{ve}$$

Finally, the model’s objective function combines the supervised hyperedge prediction loss $L_{pred}$ (main task) described in Section 4.3 with the self-supervised contrastive loss (auxiliary task) presented in this section, balanced by hyperparameter β:

(34) $$L_{total}=L_{pred}+\beta \cdot L_{ssl}$$

Through the joint optimization of these dual objectives, our model effectively utilizes the rich self-supervised signals from adaptively augmented views to alleviate data sparsity in hypergraphs, consequently improving both representation learning and generalization performance for hyperedge prediction. The contrastive strategy proposed in this subsection shares parameters with the primary hypergraph encoder during end-to-end training, ensuring information complementarity and maintaining internal model consistency.

Consequently, the OFSH synergistically tackles two fundamental challenges in hyperedge prediction via its hypergraph attention aggregator and augmentation strategy: (1) enabling accurate aggregation of node–hyperedge dynamic relationships through fine-grained influence modeling and (2) effectively mitigating data sparsity and complementing structural information acquisition via triple contrastive loss-based self-supervised learning.

## 5. Experiments and Result Analysis

### 5.1. Dataset

In this study, we utilized five distinct datasets spanning multiple academic domains: three co-citation networks (Citeseer, Cora, and PubMed), an authorship graph (Cora-A), and a collaborative network (DBLP). Notably, all datasets follow the same processing protocol as referenced in prior work [8].

- **Co-citation datasets**. Co-citation datasets model relationships through hypergraphs where nodes represent academic papers and hyperedges correspond to sets of papers co-cited by a source paper. Node features are constructed using bag-of-words representations derived from paper abstracts to capture semantic content. Three benchmark datasets are employed: Citeseer (1457 nodes, 1078 hyperedges), Cora (1434 nodes, 1579 hyperedges), and PubMed (3840 nodes, 7962 hyperedges), with feature dimensions of 3703, 1433, and 500, respectively. (https://linqs.soe.ucsc.edu/data accessed on 21 November 2024.)
- **Authorship datasets**. In authorship datasets, hypergraphs are structured such that nodes represent papers while hyperedges denote sets of papers co-authored by individual researchers. Node features are derived from bag-of-words representations of paper abstracts, with the Cora-A (https://people.cs.umass.edu/mccallum/data.html accessed on 28 November 2024.) dataset (containing 2388 nodes and 1072 hyperedges) featuring node dimensions of 1433.
- **Collaboration dataset**. This collaboration dataset uses a hypergraph model where nodes represent researchers and hyperedges denote groups of co-authors per publication. Node features are averages of bag-of-words vectors from paper abstracts authored by each researcher. The DBLP (https://lfs.aminer.cn/lab-datasets/citation/DBLP-citation-network-Oct-19.tar.gz accessed on 11 December 2024.) dataset contains 15,639 researcher nodes and 22,964 hyperedges (derived from 22,964 publications across 87 venues), with 4543-dimensional features available via the Aminer network.

Additional experimental parameters and corresponding results are comprehensively summarized in Table 1.

For the training data setup, we adopt the same configuration as [8]. Each dataset is partitioned into five distinct splits, with a 60% training set allocation and 20% each for validation and testing sets. To comprehensively evaluate the capability of OFSH, we employ four negative sampling strategies (detailed in Section 4.3) to generate negative samples matching the quantity of positive examples. These negative samples are subsequently incorporated into the training, validation, and test sets at a 1:1 ratio with positive instances for experimental validation.

### 5.2. Baselines

This study examines the performance of our newly introduced framework by benchmarking it against several leading techniques in heterogeneous network embedding. The results of this comparative analysis are summarized in Table 2.

- **Expansion** [7]. Expansion predicts future hyperedges by transforming the hypergraph into multiple n-projected graphs.
- **Hyper-SAGNN** [29]. HyperSAGNN employs a self-attention-based graph neural network model to learn candidate sets of hyperedges with variable sizes and estimates their formation probabilities.
- **NHP** [10]. NHP utilizes a hyperedge-aware graph convolutional network to learn node embeddings and aggregates the features of candidate hyperedge nodes through max–min pooling.
- **AHP** [8]. AHP combines adversarial training to generate negative samples and adopts max–min pooling for node aggregation.
- **CASH** [25]. CASH addresses the node aggregation challenge via a context-aware aggregation strategy and mitigates data sparsity through dual contrastive loss coupled with hyperedge-aware enhanced self-supervised learning.
- **OFSH.** Our proposed method.

To ensure consistency, we directly applied the findings for adversary techniques documented in [8], given that our evaluation adheres to identical assessment methodologies and employs the same partitions of datasets.

### 5.3. Implementation Details

During the experimental phase, we utilize the Adam optimization algorithm to train the OFSH framework. The hyperparameters include the following: 0.01 learning rate, 0.001 weight decay coefficient, and 0.5 dropout probability. All remaining parameters receive their initial values through Xavier’s initialization method. For all datasets, we set the batch size to 64 to fully utilize GPU memory, while maintaining the dimensionality of both node and hyperedge embeddings at 512, as specified in [8,9]. For the self-supervised learning component, we set the influence-balancing factor α to 0.7 and truncation probability to 0.8 and systematically adjust the control factor β for self-supervised loss from 0.0 to 1.0 with a step size of 0.1. Additionally, we vary both the hyperedge node masking rate $p_{node}$ and node feature masking rate $p_{f}$ across all datasets from 0.1 to 0.8 in 0.1 increments, with detailed analysis to be presented in Section 5.7.

All experiments were conducted on the following hardware configuration: Operating System: Windows 11 × 64; GPU: NVIDIA GeForce RTX 4060; CPU: Intel Core i9-13900HX; and RAM: 32GB. Our implementation of OFSH was built on PyTorch 2.2.2+cu121 and Deep Graph Library (DGL) 2.0.0+cu121.

### 5.4. Performance on Embedding

In this section, we evaluate the trained model on the test set to obtain the accuracy of hyperedge prediction, with the results presented in Table 2.

Table 2 demonstrates the performance of all competing methods on five real-world hypergraphs. The results show that our proposed OFSH consistently achieves optimal or suboptimal performance across all datasets compared to all baseline methods. Moreover, OFSH maintains the best average performance in terms of both average AUROC and average AP, indicating its balanced and superior overall capability. Specifically, on the Citeseer dataset, OFSH improves the average AUROC by 48.9%, 85.3%, 17.2%, 6.8%, and 5.0% over Expansion, HyperSAGNN, NHP, AHP, and CASH, respectively, while the AP gains are 23.9%, 64.8%, 12.4%, 3.1%, and 2.1%, respectively. Furthermore, we observe that the improvements of OFSH over CASH are significant, suggesting that our proposed hypergraph attention aggregator enhances the model’s ability to effectively capture node features and higher-order information in hypergraphs, leading to improved performance. Compared to NHP, our model exhibits superior overall performance, indicating that the hypergraph augmentation module effectively leverages the structural patterns and information within hypergraph topologies. This module facilitates better generalization by thoroughly mining critical structural information. Thus, these results demonstrate that OFSH successfully addresses the two challenges we identified in hyperedge prediction, further boosting performance by comprehensively exploiting both node features and topological information.

Although OFSH occasionally underperforms NHP and AHP in the simple negative sampling (SNS) scenario, it still achieves highly competitive AUROC scores of 96.7%, 93.8%, and 97.3% on Citeseer, Cora, and Cora-A, respectively (the best in most cases). Further analysis reveals that competing methods exhibit significantly lower accuracy in the challenging negative sampling (CNS) dataset (close to random guessing, i.e., 0.5), while performing well on the SNS test set. This substantial performance gap between SNS and CNS suggests that these methods may suffer from overfitting toward simple samples, thereby hindering their generalization across diverse datasets. In other words, these approaches fail to tackle the aforementioned two challenges and thus struggle to accurately capture higher-order interactions within hyperedges.

### 5.5. Analysis Experiment

#### 5.5.1. Attention Aggregation Analysis

In this experiment, we systematically investigated the relationship between node degrees within hyperedges and the aggregated attention weights obtained through the Member-Aware Hypergraph Attention Aggregator (MHAA). To intuitively visualize this correlation, we employed bubble charts where both the size and color intensity of the bubbles are proportional to the values of node degrees or attention weights—larger values correspond to bigger bubble sizes and darker colors, with this dual encoding scheme effectively enhancing the intuitiveness of the visualization.

**Figure 5:**
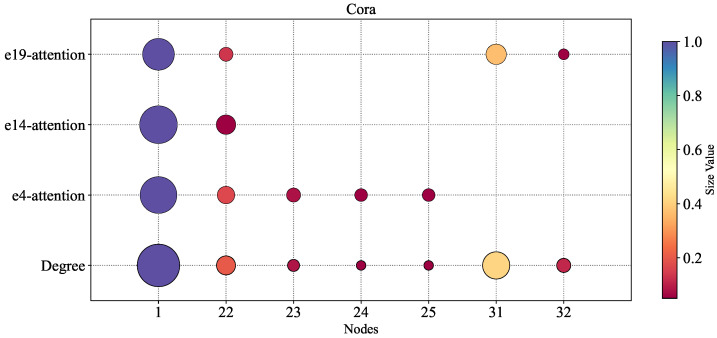
The relation between node degrees and aggregation attention in a hyperedge. The horizontal direction represents the sequence number of the nodes.

For simplicity of analysis, we selected three distinct hyperedges ($e_{4}$, $e_{14}$, and $e_{19}$) to which the node $v_{22}$ belongs, examining the distribution of node degrees and attention weights within them. As shown in Figure 5, a significant positive correlation exists between node attention weights and node degrees across all three hyperedges. This indicates that nodes with higher degrees receive greater attention weights in the MHAA module, while lower-degree nodes obtain relatively less attention. This finding validates that the MHAA mechanism can effectively capture topologically central nodes within the network (i.e., core members with high influence), thereby improving the model’s representation capacity of hypergraph structures through precise allocation of attention weights, ultimately enhancing the accuracy of hyperedge prediction.

#### 5.5.2. Order Attention Effectiveness Analysis

**Figure 6:**
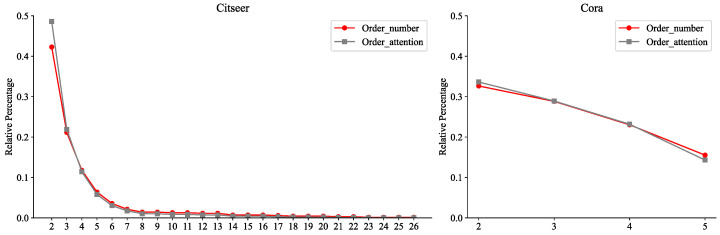
Evaluation of the difference between real order counts and average order attention in MNS.

To systematically validate the effectiveness of the order attention mechanism in our proposed OHAA module, we conducted a quantitative comparison between the actual counts of hyperedges across different orders and the corresponding average attention scores derived during node training. For instance, the average four-order attention was computed by aggregating attention scores from all nodes for four-order hyperedges. Results on the SNS dataset, Figure 6, reveal a statistically significant positive correlation between the node-specific order counts and their average attention weights. This observation not only empirically demonstrates the attention mechanism’s capability to precisely capture the inherent distribution patterns of higher-order structures in the dataset but also provides deeper insights into the intrinsic consistency between node attention weights and hyperedge topological features (e.g., order density). Specifically, the model actively reinforces its focus on hyperedges with higher-density orders through learning, which further corroborates the core advantage of OHAA’s design: by jointly modeling node influence and hyperedge scale effects via hierarchical attention mechanisms, OHAA effectively distinguishes the differential semantic contributions of hyperedges across varying orders. This end-to-end framework significantly enhances the model’s interpretive efficiency for hypergraph structural characteristics and their real-world distribution patterns.

#### 5.5.3. Maxmin Method Effectiveness Analysis

As detailed in Table 3, we systematically evaluate three feature extraction strategies within the OHAA module for the hyperedge prediction task, focusing specifically on order-specific features from node neighborhood hyperedges. Our experimental results demonstrate that max–min pooling significantly outperforms both summation and averaging strategies. The performance advantage of max–min pooling validates the theoretical analysis presented in Section 4.2.2: it preserves the intra-hyperedge group heterogeneous structures of the same order through feature range retention, while preventing the dilution of discriminative signals caused by sum/mean operations.

This superiority stems from its ability to handle dominant hyperedges—those that frequently recur or exhibit disproportionately salient features within a given order. While summation or averaging would homogenize order-specific features across nodes, potentially reducing discriminative power, max–min pooling effectively computes the difference between maximum and minimum feature values per dimension. This approach simultaneously preserves (1) the most representative patterns from dominant hyperedges and (2) distinctive variations from non-dominant hyperedges. Consequently, it amplifies order-specific feature distinctiveness and enhances the model’s capacity to discern each node’s unique structural context.

#### 5.5.4. Training Time

**Figure 7:**
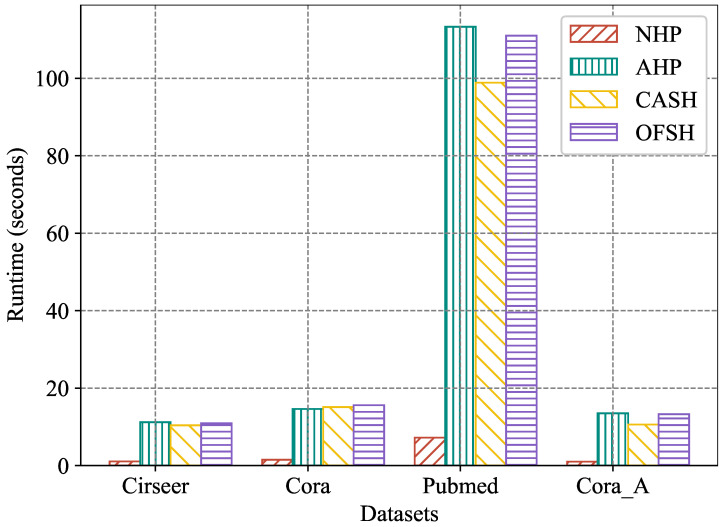
Running time (seconds) with 5 epochs.

In this experiment, we employed four real-world hypergraph datasets to compare the training efficiency of OFSH and other baseline methods. Specifically, we selected three competitive approaches—NHP, AHP, and CASH—and trained them under a unified environment, recording the time required to complete five training epochs for each method. For all compared methods, the official code released by the original authors was used, and hyperparameter settings were kept consistent throughout the experiments. Figure 7 illustrates the average running time of each method across the five training epochs. The results show that, although OFSH requires slightly more time to complete training compared to CASH, it is faster than AHP. Considering that OFSH consistently outperforms both CASH and AHP across all datasets and all hyperedge prediction accuracy metrics, these results indicate that OFSH can more effectively model higher-order interactions in real-world scenarios with comparable model complexity. Although NHP requires substantially less training time than OFSH, its hyperedge prediction accuracy is markedly inferior to that of CASH.

### 5.6. Ablation Experiment

In this experiment, we conduct ablation studies to better analyze the influence of each component in our OFSH framework on embedding performance, examining the following variants:

1. No hypergraph Attention Aggregator and no Hypergraph Augmentation (NOAA-HA): This approach eliminates both the hypergraph attention aggregation module and removes the self-supervised contrastive loss for hypergraph augmentation.
2. No hypergraph Attention Aggregator with Random augmentation (NOAA-Random): This variant removes the hypergraph attention aggregation module and replaces the importance-based masking augmentation with purely random augmentation while retaining the self-supervised contrastive loss in training.
3. No hypergraph Attention Aggregator (NOAA): It removes the hypergraph attention aggregation module while retaining the hypergraph augmentation module proposed in this work and incorporates the self-supervised contrastive loss into the training process.

The hyperedge prediction performance of OFSH and its three model variants is presented in Table 4. Overall, each strategy we proposed contributes to improving the accuracy of the OFSH model. Specifically, when all strategies are applied to OFSH, the average AUROC shows improvements of 10.56%, 13.90%, and 9.03% compared to NOAA-HA on Citeseer, Cora, and Cora-A datasets, respectively, while the average AP improves by 6.97%, 12.74%, and 7.26%.

NOAA-Random outperforms NOAA-HA across all test sets of the evaluated datasets. This result validates that the self-supervised contrastive learning module introduced in OFSH can more effectively utilize hypergraph topological information, deeply mine auxiliary information through the contrastive learning mechanism, and successfully alleviate data sparsity issues. Furthermore, the average AUROC of NOAA surpasses that of NOAA-Random, demonstrating NOAA’s superior hyperedge prediction capability. This indicates that our proposed hypergraph augmentation strategy—which generates augmented views adaptively while preserving original structural properties through key nodes and feature importance—significantly outperforms simple random augmentation approaches and can generate enhanced views adaptively according to different hypergraphs’ structural characteristics. Finally, across all datasets, OFSH achieves higher average AUROC than NOAA, confirming that our hypergraph attention aggregator can effectively capture both node features and high-order structural information.

### 5.7. Hyperparameter Study

In this experiment, we analyze the hyperparameter sensitivity of OFSH.

**Figure 8:**
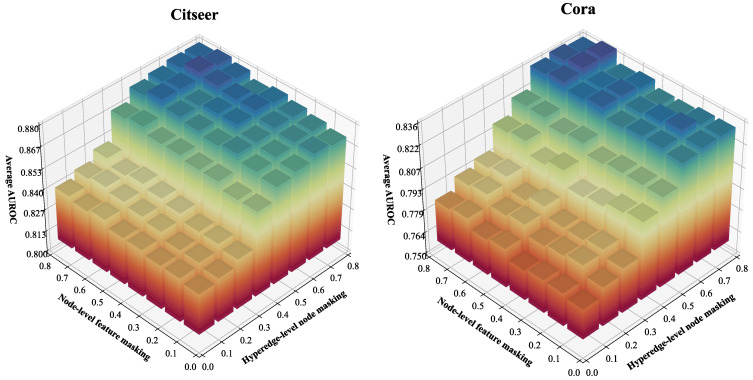
Analyzing the impact of hyperedge-level node and node-level feature masking rates $p_{node}$ and $p_{f}$.

First, we investigate the impact of masking probability hyperparameters $p_{node}$ and $p_{f}$ on OFSH’s predictive performance. As described in Section 4.4, hyperparameter $p_{node}$ regulates the global masking ratio of nodes within each hyperedge in the augmented view, while hyperparameter $p_{f}$ controls the overall masking probability at the node feature dimension level. As $p_{node}$ and $p_{f}$ increase, the masking probabilities for both hyperedge nodes and feature dimensions in the augmented view also rise. We evaluated OFSH’s AUROC accuracy by varying $p_{node}$ and $p_{f}$ in the interval [0.1, 0.8] with a step size of 0.1. The experimental results are presented in Figure 8, where the x-axis represents $p_{node}$, the y-axis represents $p_{f}$, and the vertical axis denotes the average AUROC. The results demonstrate that when $p_{node}\geq 0.4$, the average AUROC rises significantly and maintains high accuracy across a broad range of values. Conversely, when $p_{node}<0.4$, OFSH’s hyperedge prediction accuracy is generally poor. Additionally, when both $p_{node}$ and $p_{f}$ are set to 0.1 (i.e., when the hypergraph augmentation module’s effect is minimal), the model performs among the worst. Furthermore, the variation in average AUROC with respect to $p_{node}$ is substantially greater than that with respect to $p_{f}$. These findings imply the following: (1) In contrastive learning aimed at capturing the structural information of the original hypergraph, hyperedge-level topology augmentation plays a more critical role in improving model performance than node-level feature augmentation. (2) OFSH achieves high accuracy across a wide range of hyperparameter values.

**Figure 9:**
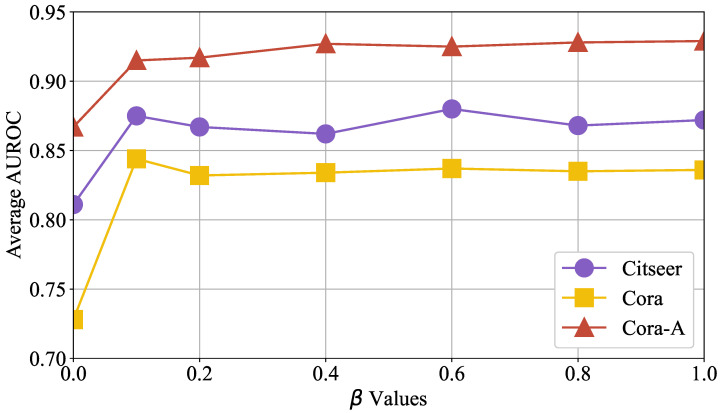
The influence of self-supervised contrastive learning on OFSH’s hyperedge prediction performance varies with the control hyperparameter β.

Next, we evaluate the influence of self-supervised contrastive learning on OFSH’s accuracy. We measured its performance on three distinct test sets while gradually increasing the loss control factor β from 0.0 to 1.0 in increments of 0.1. The experimental results, presented in Figure 9, show the x-axis as β and the y-axis as the average AUROC of training accuracy. When β exceeds 0.1, the model’s accuracy improves significantly across all test sets and remains high over a broad parameter range. This suggests the following: (1) Self-supervised contrastive learning consistently enhances OFSH’s prediction performance by supplementing the learning of high-order hyperedge information encoding. (2) Once β surpasses a certain threshold, OFSH’s accuracy becomes insensitive to its variation.

## 6. Conclusions

We proposes the Order propagation Fusion Self-supervised learning for Hyperedge prediction (OFSH) framework to address challenges in hyperedge prediction, including node aggregation and hyperedge augmentation. Key innovations include the following: (i) A dual-attention mechanism comprising the Member-Aware Hypergraph Attention Aggregator (MHAA), which dynamically weights node importance within hyperedges, and the Order-Specific Hyperedge Attention Aggregator (OHAA), which groups hyperedges by order and applies max–min pooling to amplify feature distinctions. OHAA further employs cross-order attention propagation to jointly model node influence and hyperedge scale effects, significantly enhancing semantic discrimination. (ii) A key node-guided augmentation strategy leveraging topological importance metrics and adaptive masking probabilities, preserving core high-order semantics while generating robust contrastive views. (iii) A tri-level contrastive loss operating at node, hyperedge, and association levels to maximize cross-view consistency and mitigate sparsity.

The performance breakthrough of OFSH gives it significant value in practical applications that rely on higher-order relationship modeling. In recommendation systems, OFSH’s precise higher-order hyperedge prediction capability enables accurate group recommendation scenarios, including (1) E-commerce co-purchase prediction—identifying product groups frequently bought together by similar user segments; (2) social circle detection—predicting potential interest-based user communities in social networks; and (3) content bundle recommendation—recommending complementary content packages. Its dynamic node influence modeling effectively identifies core group members and influencers, thereby improving recommendation accuracy and diversity. In bioinformatics, OFSH shows particular promise for (1) protein complex prediction—accurately identifying molecular complexes in protein–protein interaction networks by capturing intricate multi-protein interactions; (2) drug combination prediction—predicting synergistic drug combinations for combinatorial therapy; and (3) functional module discovery—identifying coherent functional modules in biological networks. The adaptive key node masking strategy ensures robustness against noisy biological data, while the order-specific attention mechanism captures subtle relationship patterns in molecular interactions.

## Figures and Tables

**Table 1:** Details of hyperedge datasets.

Datasets	Category	$\left|V\right|$	$\left|E\right|$	#Features	Ave.Order	Max.Order	Min.Order
Citeseer	Co-citation	1457	1078	3703	3.2	26	2
Cora	Co-citation	1434	1579	1433	3	5	2
PubMed	Co-citation	3840	7962	500	4.3	171	2
Cora-A	Authorship	2388	1072	1433	4.3	43	2
DBLP	Collaboration	15,639	22,964	4543	2.7	18	2

**Table 2:** Quantitative results on hyperedge prediction task (%). Bold for “the best” and underline for “the second best”.

Datasets	Metrics	AUROC					Average Precision (AP)				
	Test Set	SNS	CNS	MNS	MIX	Average	SNS	CNS	MNS	MIX	Average
**Citseer**	Expansion	0.663	0.331	0.781	0.558	0.591	0.765	0.498	0.871	0.631	0.681
	HyperSAGNN	0.540	0.473	0.410	0.478	0.475	0.627	0.497	0.455	0.507	0.512
	NHP	**0.991**	0.510	0.701	0.817	0.751	**0.990**	0.520	0.731	0.768	0.751
	AHP	0.943	0.651	0.881	0.820	0.824	0.952	0.660	0.870	0.795	0.819
	CASH	0.928	0.708	0.906	0.838	0.838	0.927	0.705	0.910	0.824	0.827
	**OFSH**	0.967	**0.744**	**0.935**	**0.879**	**0.880**	0.969	**0.714**	**0.922**	**0.838**	**0.859**
**Cora**	Expansion	0.470	0.256	0.707	0.476	0.477	0.637	0.454	0.764	0.563	0.607
	HyperSAGNN	0.617	0.494	0.527	0.540	0.545	0.687	0.508	0.574	0.566	0.584
	NHP	0.943	0.472	0.641	0.774	0.703	0.949	0.509	0.678	0.744	0.718
	AHP	**0.964**	0.572	0.860	0.799	0.799	**0.961**	0.552	0.837	0.740	0.772
	CASH	0.918	0.642	0.857	0.812	0.811	0.913	0.617	0.846	0.771	0.784
	**OFSH**	0.938	**0.684**	**0.869**	**0.844**	**0.836**	0.935	**0.660**	**0.870**	**0.829**	**0.823**
**PubMed**	Expansion	0.520	0.241	0.730	0.497	0.497	0.675	0.440	0.755	0.565	0.612
	HyperSAGNN	0.525	0.546	0.686	0.580	0.584	0.534	0.529	0.680	0.561	0.576
	NHP	**0.973**	0.524	0.694	0.745	0.733	**0.973**	0.513	0.656	0.678	0.707
	AHP	0.917	0.553	0.840	0.763	0.763	0.918	0.526	0.834	0.717	0.749
	CASH	0.801	0.636	0.867	0.769	0.774	0.804	0.639	0.875	0.761	0.764
	**OFSH**	0.849	**0.646**	**0.879**	**0.776**	**0.786**	0.852	**0.648**	**0.886**	**0.766**	**0.788**
**Cora-A**	Expansion	0.690	0.434	0.842	0.658	0.656	0.690	0.577	0.876	0.672	0.706
	HyperSAGNN	0.386	0.542	0.591	0.505	0.506	0.532	0.545	0.643	0.563	0.571
	NHP	0.909	0.550	0.672	0.773	0.723	0.925	0.585	0.720	0.766	0.748
	AHP	0.958	0.782	0.924	0.887	0.888	0.957	0.796	0.898	0.878	0.882
	CASH	0.954	0.794	0.956	0.906	0.905	0.955	0.794	0.956	0.900	0.901
	**OFSH**	**0.973**	**0.834**	**0.972**	**0.940**	**0.930**	**0.975**	**0.839**	**0.972**	**0.934**	**0.931**
**DBLP**	Expansion	0.645	0.366	0.801	0.607	0.607	0.751	0.518	0.856	0.655	0.698
	HyperSAGNN	0.448	0.572	0.574	0.530	0.531	0.562	0.586	0.602	0.577	0.582
	NHP	0.663	0.503	0.540	0.572	0.569	0.608	0.501	0.523	0.542	0.544
	AHP	**0.946**	0.568	0.820	0.778	0.778	**0.947**	0.561	0.815	0.735	0.764
	CASH	0.840	0.701	0.826	0.789	0.791	0.839	0.687	0.816	0.779	0.782
	**OFSH**	0.884	**0.713**	**0.838**	**0.813**	**0.811**	0.878	**0.693**	**0.833**	**0.798**	**0.800**

**Table 3:** Assessment of various order feature extraction methods for hyperedge prediction. Bold for “the best”.

Datasets	Extraction Strategies	AUROC	Average Precision (AP)
Citseer	Sum	0.953	0.955
	Mean	0.952	0.953
	Max–min	**0.967**	**0.969**
Cora	Sum	0.928	0.927
	Mean	0.929	0.926
	Max–min	**0.938**	**0.935**
Cora-A	Sum	0.963	0.962
	Mean	0.961	0.963
	Max–min	**0.973**	**0.975**

**Table 4:** Impact of the suggested methods on enhancing model prediction precision. Bold for “the best”.

Datasets	Metrics	AUROC					Average Precision (AP)				
	Test Set	SNS	CNS	MNS	MIX	Average	SNS	CNS	MNS	MIX	Average
**Citseer**	NOAA-HA	0.882	0.645	0.859	0.798	0.796	0.895	0.670	0.862	0.788	0.803
	NOAA-Random	0.910	0.685	0.898	0.836	0.833	0.912	0.683	0.879	0.822	0.824
	NOAA	0.913	0.698	0.905	0.842	0.840	0.915	0.702	0.889	0.828	0.833
	**OFSH**	**0.967**	**0.744**	**0.935**	**0.879**	**0.880**	**0.969**	**0.714**	**0.922**	**0.838**	**0.859**
**Cora**	NOAA-HA	0.866	0.560	0.780	0.732	0.734	0.864	0.563	0.776	0.720	0.730
	NOAA-Random	0.909	0.620	0.857	0.795	0.795	0.883	0.589	0.829	0.765	0.766
	NOAA	0.911	0.625	0.863	0.799	0.799	0.890	0.591	0.831	0.781	0.780
	**OFSH**	**0.938**	**0.684**	**0.869**	**0.836**	**0.836**	**0.935**	**0.660**	**0.870**	**0.829**	**0.823**
**Cora-A**	NOAA-HA	0.949	0.703	0.905	0.853	0.853	0.950	0.746	0.910	0.867	0.868
	NOAA-Random	0.945	0.766	0.937	0.889	0.885	0.948	0.783	0.939	0.893	0.890
	NOAA	0.972	0.832	0.951	0.923	0.920	0.972	0.847	0.921	0.910	0.912
	**OFSH**	**0.973**	**0.834**	**0.972**	**0.940**	**0.930**	**0.975**	**0.839**	**0.972**	**0.934**	**0.931**

## Data Availability

The research data will be shared when accepted.
